# Healthcare Services for Asylum-Seekers: Untangling the European Social Charter

**DOI:** 10.1093/medlaw/fwad018

**Published:** 2023-07-06

**Authors:** Yana Litins’ka

**Affiliations:** Department of Law, Lund University; Senior Lecturer in Public Law, Lund, Sweden

**Keywords:** Asylum-seekers, European Social Charter, Healthcare services, Right to health, Right to medical assistance

## Abstract

Asylum-seekers, like any population, need healthcare services, yet national laws sometimes restrict access to such services. The European Social Charter (revised) protects the right to health and medical services. However, the Charter has a complex application, and its scope is limited concerning foreigners. This article analyses to what extent the provisions of the Charter on the right to health and medical assistance apply to adult asylum-seekers. It shows that the Charter may apply to various degrees to asylum-seekers depending on several circumstances, such as the national definition of residence or regular work, grounds for seeking asylum, citizenship or lack thereof. Depending on these factors, some asylum-seekers may receive full healthcare services, whereas others may have only limited rights. The article shows that the migrant statuses created by national and EU law do not fit in the system of statuses in the Charter, which might produce legal hindrances to accessing health-related rights for asylum-seekers. The article also discusses the possible ways for the European Committee of Social Rights to further expand the scope of the Charter’s application.

## I. BACKGROUND AND AIM

Being an asylum-seeker means having a temporary status: it will change when authorities decide to reject the application or guarantee international protection. Yet people often live with this status for years. While waiting for the authorities’ decision, asylum-seekers can get sick or continue being ill. Many circumstances before, during, or after migration may elevate asylum-seekers health risks, which should alert about the need for healthcare services.^1^

The European regulation provides complex responses as to what healthcare shall be offered to asylum-seekers, where European Union (EU), international human rights, and national laws interact. EU law obliges Member States to guarantee asylum-seekers the minimum standard of access to necessary healthcare, including emergency care and essential treatments.^2^ However, most EU states decided not to grant full access to healthcare services, above the minimum requirement of the EU law.^3^ The national laws often hinder asylum-seekers access to various medical services that are not considered an emergency or strictly essential but valuable from individual or public health perspectives. These non-emergency services include preventive screenings or treatments, such as cancer screenings or vaccinations. The existing national legislation leads to civic stratification—the differentiation between the rights of citizens and various categories of non-citizens—in enjoyment of the rights and accessing welfare.^4^ The vulnerability of asylum-seekers and the co-existence of multiple legal orders necessitate advancing the understanding of how human rights treaties protect access to healthcare services for asylum-seekers in Europe and relate to civic stratification.

Within the Council of Europe, the most comprehensive protection of access to healthcare services is granted by the system of the European Social Charter.^5^ Several rights in the Charter concern accessibility of healthcare services; among them are the rights to health and medical assistance. Although the Charter has been in force for a long time, its provisions on health rights for asylum-seekers have been insufficiently studied. Unlike other Council of Europe treaties establishing the rights to health or medical assistance,^6^ the Charter’s system sets up a mechanism for monitoring the states’ fulfilment of their obligations, including through collective complaints (through the additional protocol to the Charter). These functions are laid upon the European Committee of Social Rights (ECSR or Committee), a quasi-judicial body that creates case law interpreting the Charter.

This article aims to analyse to what extent the European Social Charter (revised) (ESCr) provisions on the right to health and medical assistance apply to adult asylum-seekers.^7^ The analysis will show that asylum-seekers may be attributed to different healthcare rights under the ESCr depending on citizenship, national definitions of residence and regular work and reasons for seeking asylum. These differences imply obstacles in navigating the ESCr for decision-makers and asylum-seekers. The article will suggest ways for the Committee to reconcile some difficulties in applying the Charter.

In the analysis, particular weight will be given to the supplementary interpretation sources of the ESCr. These include preparatory works, the explanatory report, and case practice of the Committee. In the article, conclusions based on state reports, decisions on collective complaints and statements of interpretations will be analysed to arrive at conclusions as to the applicability of the treaty and the substance of the rights.

## II. THE CATEGORIES OF FOREIGNERS IN THE CHARTER

The ESCr was not designed as a universal instrument applicable to everyone: the treaty has limited personal scope regarding non-citizens of state parties. The provisions indicating the limitations of the personal scope are laid down in the Appendix, which is the consistent part of the Charter. Such a design can be explained by the history of the Charter creation. It was initially constructed to be an instrument for ensuring reciprocity in fulfilling the duties of the states.^8^ The preparatory works point out that the Charter aimed to protect against discrimination based on the nationality of the residents of the state parties only.^9^ Additionally, since the rights in the Charter relate to social insurance systems that may be different in each state party, the Charter narrowed the scope to the residents because they were considered to have access to such insurance systems.^10^

Paragraph 1 of the Appendix to the ESCr lays down the general rule of the Charter’s application, namely, the treaty applies to foreigners only if two conditions are fulfilled simultaneously. The first condition is that foreigners shall be recognised as lawful residents, alternatively, regularly working in a state. The second condition is that these foreigners must be nationals of the contracting party of the Charter.

The ESCr’s Appendix establishes several exemptions from the general rule introduced above. Some of these exceptions are addressed to specific groups of foreigners. In particular, paragraph 2 of the ESCr’s Appendix determines that the treaty shall apply to refugees as defined in the Convention relating to the Status of Refugees (Refugee Convention).^11^ Furthermore, paragraph 3 of the Appendix sets forth that the Charter applies to stateless persons as defined in the Convention on the Status of Stateless Persons.

In contrast with paragraph 1 of the Appendix, the requirement of being a national of the ESCr’s Member State is not stated for the refugees and stateless persons. These groups also do not need to be lawful residents or regular workers. However, they must lawfully stay on the territory of a state. States shall guarantee at least treatment for refugees and stateless persons on the level defined in the Refugee Convention and the Convention on the Status of Stateless Persons. This level depends on the right in question; it is determined through reference to a comparative group. Refugees and stateless persons shall be treated at the same level as citizens, other aliens generally, and foreigners who receive the most favourable treatment. The ECSR—the monitoring body of the ESCr—recognizes that the states must guarantee the right to health (Article 11 of the ESCr) and the right to medical assistance (Article 13 of the ESCr) at the same level as citizens to refugees and stateless persons.^12^

Paragraph 1 of the Appendix to ESCr also reminds states about four Charter provisions that may establish a different scope of their application. These provisions concern the right to social security (Article 12(4)), to engage in a gainful occupation (Article 18), the rights of migrant workers and their families to protection and assistance (Article 19), and, most important for this paper, the right to medical assistance (Article 13(4)).

All these mentioned provisions refer to different groups of foreigners: Article 12(4) of the ESCr concerns the exportability of social benefits for those foreigners who were previously lawful residents and nationals of a state party. Article 18 of the ESCr provides guarantees related to the right to engage in a gainful occupation for those foreigners who are residents of the other party to the Convention, however, not residents in a specific Member State. Article 19 expands the scope of protection for members of the family of migrant workers in cases of their departure, journey, and several weeks upon arrival. These conditions also concern the possibility of receiving healthcare services but only while travelling or shortly after arrival. Thus, the personal scope of the Convention covers family members who are not residents but are present on the territory with migrant workers that fulfil the criteria for applicability. Finally, Article 13(4) of the ESCr guarantees the right to social and medical assistance to foreigners who are lawfully staying within the territory of a Member State. The provision means that the first general condition—being a resident or regular worker—established in the Appendix is not applicable. However, the second condition of being a national to a state party applies.^13^

Thus, the treaty text emphasies the difference between residents and persons lawfully staying within the territory that the Charter’s text makes. Yet, the Charter contains no definitions of residency, lawful stay or regular work. It also does not provide references to other treaties that define these terms. So far, the ECSR has not attempted to develop autonomous definitions of these categories. It can be assumed that the ESCr, with its subsidiary functions to national legislation, relies on the domestic law definitions of these categories.

To sum up, the overview of the provisions of the Appendix to the ESCr indicates that the treaty yields civic stratification via distinguishing between citizens and several groups of foreigners. Civic stratification also exists between different groups of foreigners because they are able to enjoy the protection of the Charter to various degrees. These groups of foreigners and the literal application of the Charter towards them are illustrated in Table 1 below.

This article will further refer to this numbered classification of the groups to avoid cluttering. The following discussion will not concentrate on groups 3–5 but on healthcare services for groups 1, 2, 6–9. Asylum-seekers can be seen as group 5, but relatively rarely; the discussion on the normative substance of Article 19 ESCr is necessarily precluded.

**Table 1.** Groups of foreigners within the Charter

Group number in this article	Group of foreigners in the Charter	Granted rights in the ESCr following the literal interpretation
Group 1	Status: Foreigners unlawfully staying within a state party (irregular migrants)	Excluded from the scope
	Citizenship: Any	
Group 2	Status: Lawfully staying or residing or regularly working foreigners	Excluded from the scope
	Citizenship: Non-citizens of a state party	
Group 3	Status: Foreigners that seek gainful occupation	The right to engage in a gainful occupation (partly)
	Citizenship: Citizens of another state party	
Group 4	Status: Foreigners that used to be lawful residents	The right to social security (exportability of social benefits)
	Citizenship: Citizens of a state party	
Group 5	Status: Lawfully staying foreigners that are members of the family of the migrant worker, who is residing legally	The right to assistance during the journey, departure and reception
	Citizenship: Migrant workers shall be citizens of a state party	
Group 6	Status: Lawfully staying foreigners	The right to social and medical assistance
	Citizenship: Citizens of a state party	
Group 7	Status: Lawfully staying refugees as defined in the Refugee Convention	The right to health and the right to social and medical assistance at the same level as nationals. The scope of other rights is determined through references to comparative groups
	Citizenship: Any	
Group 8	Status: Lawfully staying stateless persons as defined in the Convention on the Status of Stateless Persons	The right to health and the right to social and medical assistance at the same level as nationals. The scope of other rights is determined through references to comparative groups
	Citizenship: None	
Group 9	Status: Lawfully residing or regularly working foreigners	At the same level as nationals, including the right to health and the right to social and medical assistance
	Citizenship: Citizens of a state party	

## III. HEALTH-RELATED RIGHTS IN THE CHARTER: WHAT MAKES THEM DIFFERENT?

### A. The right to medical assistance

In Section II, it was clarified that in the Charter, different groups of foreigners are entitled to various catalogues of rights. Literal interpretation entitles groups 7–9 to both the right to health and medical assistance, whereas group 6 is granted the right to medical assistance only. These variations beg to answer how the material scopes of the rights to health and medical assistance differ.

Section III will analyse the material scope of the rights to medical assistance and health. Section A provides introductory remarks but focuses on the material scope of the right to medical assistance. The right to health will be spotlighted in Section B.

Everyone’s enjoyment of the highest possible standard of health that is attainable and medical assistance for those in need are set among the objectives of the treaties in Part I of the Charter. The fact that these values are set as objectives is essential, in particular, for the teleological interpretation of the treaty.^14^

The rights established in the ESCr do not have an absolute character. The possibility of limiting the rights is foreseen in Article G ESCr. Limitations in Article G concern all the rights established in the Charter, including the right to health and medical assistance. The limitations of the rights are lawful when prescribed by law, have a legitimate purpose (such as protecting the rights and freedoms of others, the public interest, national security, public health, or morals) and are necessary in a democratic society. However, the Committee has not yet discussed the limitations in case law on the right to health and medical assistance.

The focus will now be on the right to medical assistance in Article 13. Article 13(1) of the ESCr reads as follows:

With a view to ensuring the effective exercise of the right to social and medical assistance, the Parties undertake:

1. to ensure that any person who is without adequate resources and who is unable to secure such resources either by his own efforts or from other sources, in particular by benefits under a social security scheme, be granted adequate assistance, and, in case of sickness, the care necessitated by his condition.

The provisions of Article 13(1) ESCr are formulated as having an immediate realisable nature. They require the states to show concrete results, emphasised by the wording ‘ensure’ and ‘be granted’.^15^ The Committee also stresses that the right enshrined is connected with the principle of human dignity and has an immediately realisable nature: the obligations of the states arise when a person is in need.^16^

The provisions of Article 13(1) ESCr are activated when a person lacks adequate resources, suggesting that the Article is primarily concerned with the economic accessibility of medical assistance. The Article does not directly determine the scope of medical services a state must grant. The obligations to provide medical assistance are not dependent on a person’s previous contributions to the social security schemas or budget.^17^

Suppose the costs of medical assistance are not affordable. In that case, Article 13(1) ESCr requires assisting in kind, referred to in the Article as ‘care necessitated’ by a medical condition or in cash, to make treatment affordable, referred to as ‘adequate assistance’. The Article, thus, concerns the situations when the treatment shall be provided free of charge, subsidized by a state, or a lump sum should be given to enable access to treatment.^18^

Concerning unaffordable care in kind that the states shall provide—or ‘necessitated’ care—the Committee states that this is at least immediately necessary care, such as emergency medical care, but it shall not be limited to such care.^19^ The medical assistance shall include both primary and specialised outpatient care.^20^ As to the care in cash, in its most recent conclusions on Article 13 of the Charter, the Committee requests the states to provide information about non-urgent medical assistance to uninsured persons: it asks whether the uninsured persons have the same access to healthcare as the insured.^21^ Overall, the Committee interprets the Charter as obligating the states to provide adequate medical assistance for their nationals and lawfully residing or staying citizens of other state parties on an equal basis.^22^ The conclusions also emphasise that benefits should be provided, disregarding the length of stay on the territory^23^ or misconduct committed by care seekers.^24^ Additionally, the Committee stresses that medical assistance shall not be limited in time^25^ or depend on the length of residence.^26^

To sum up, Article 13 of the ESCr establishes duties when persons lack adequate resources and, thus, are concerned with services’ economic accessibility (affordability). The Article does not specify what care shall generally be available within a state but requires guaranteeing necessary care in kind or adequate assistance in cash (providing money to pay for the care or subsidising care) for every regularly working, lawfully residing or staying national of a state party, refugee or a stateless person (groups 6–9). The Article embeds immediately realisable obligations, which are activated notwithstanding the length of stay. The state obligations are generally not limited to primary and emergency care but go beyond that.

### B. The right to health

Now the attention will turn to the right to health under Article 11 of the ESCr, which fully applies to groups 7–9. The Article reads as follows:

With a view to ensuring the effective exercise of the right to protection of health, the Parties undertake, either directly or in cooperation with public or private organisations, to take appropriate measures designed inter alia:

1. to remove as far as possible the causes of ill-health;

2. to provide advisory and educational facilities for the promotion of health and the encouragement of individual responsibility in matters of health;

3. to prevent as far as possible epidemic, endemic and other diseases, as well as accidents.

The right to health in Article 11 ESCr is formulated as a positive obligation of conduct (as opposed to the obligation of result in Article 13), emphasised by the phrasing ‘take appropriate measures’.^27^ As can be seen from the text, Article 11 of the ESCr offers a non-exhaustive list of areas where the states shall take steps to promote health. The scope of the right to health under the ESCr is broad and more expansive than the content of Article 13. The Committee contemplates that ‘health’ shall be defined in the same manner it is defined in the World Health Organization’s Constitution, namely as a ‘state of complete physical, mental and social well-being and not merely the absence of disease or infirmity’.^28^ The Article’s broad wording can explain why determinants of health (such as housing, water, food, and sanitation) are often spotlighted in the reasoning of the Committee.^29^

Although Article 11 of the ESCr does not mention healthcare services, they are relevant for all the examples of states’ actions provided in the text. For instance, when the absence of medical services can cause ill health, such as in diabetes, cancer, or (some) uterine fibroids, Article 11(1) may become relevant. The provision of psycho-social counselling or other advisory services appears to fall within the ambit of Article 11(2) ESCr. Various preventive treatments and services, such as vaccination or medical screenings, shall be a consistent part of Article 11(3) ESCr. In the first statement of interpretation of Article 11, the Committee confirmed this understanding. The Committee stated that the right to health encompasses the State’s obligations to enable access to medical care to address main health problems. These obligations shall include providing the ‘proper medical care for the whole population’ and diagnostic and preventive measures.^30^ This interpretation was further reinforced in the practice of the Committee through the years.^31^

The abovementioned reference to proper medical care appears to relate to several aspects of health service provision. Among these aspects, various facets of *accessibility* were highlighted. *Geographic accessibility* of healthcare services, particularly for the rural population, was stressed.^32^ Access to healthcare *without discrimination* has been a criterion to assess compliance with Article 11 ESCr.^33^ The Committee interpreted Article 11.2 ESCr as required to ensure *information accessibility* and *acceptability*, particularly by awareness-raising various health-related issues, treatments and diseases, and culturally appropriate education.^34^ These aspects of accessibility appear to be especially relevant for migrants, such as asylum-seekers. *Economic accessibility* of healthcare services was explicitly highlighted in the practice of the Committee, which overlaps with the scope of Article 13 ESCr.^35^ The Committee considered that the healthcare practitioners, hospital beds, and equipment shall be *available* in sufficient quantities without unnecessary delays.^36^ The Committee monitoring has also focused on the quality-related aspects of healthcare services.^37^

Thus, although the Committee does not explicitly refer to the elements of the right to health as to the availability, accessibility, acceptability, and quality, the reasoning about all these components is present in the case practice.^38^ The right to health addresses the question of what kind of care shall be available and how it should be provided so it is available, accessible, acceptable for everyone and has good quality.

## IV. PLACING ASYLUM-SEEKERS IN THE CHARTER’S SYSTEM

### A. Legal grounds for seeking asylum

The introduction of the personal scope, provided in Section II, indicates that some groups of foreigners are expressly considered in the Charter, but there are no explicit references to the asylum-seekers. In this section, the status of asylum-seekers in the ESCr will be analysed and problematised. The analysis will start with clarifying the legal grounds for seeking asylum.

As mentioned above, asylum-seekers are those persons that seek international protection but have not yet received the decision of authorities. The possibilities to receive international protection are regulated by national law, EU law (for the EU Member States, which the majority of the Council of Europe states are) and international law, such as the Refugee Convention mentioned above. The term ‘asylum-seeker’ is sometimes used to include only those that seek asylum based on the Refugee Convention, but in this article, I use it to describe persons seeking international protection based on national, EU or international law.

In the Refugee Convention, a refugee is defined as a person who, owing to a well-founded fear of being persecuted for specific reasons of race, religion, nationality, membership of a particular social group, or political opinion, is unable or unwilling to avail themselves of the protection of that country.^39^ The definition mentioned above does not protect groups fleeing from generalised violence, such as during armed conflict, as the main rule. However, since many armed conflicts endanger ethnic, religious, and other minorities, the Refugee Convention may apply to these groups if the connection with the discrimination grounds has been shown in each individual case.^40^

While EU law acknowledges the reasons for seeking asylum stated in the Refugee Convention, it provides additional possibilities for such applications in a Member State of the EU. These other possibilities are regulated in Article 15 of the Qualification Directive and are often referred to as subsidiary grounds for protection. One such ground is a threat to life or person because of indiscriminate violence in international or internal armed conflict.^41^

Similarly, the national legislation may provide additional possibilities for seeking asylum within a specific state compared to the Refugee Convention or Qualification Directive, such as obtaining necessary medical care. However, studying the list of such reasons goes beyond the scope of this article.^42^ Therefore, asylum-seekers can seek protection based on the Refugee Convention, subsidiary grounds established by Qualification Directive, if the application is made in EU states, or additional grounds provided in national law.

Suppose an asylum-seeker has claimed some grounds for protection, and authorities do not find that application qualifies for such a ground. In that case, authorities are obligated to examine whether other grounds exist. The reasons for applying for asylum, on the one hand, and granting or rejecting protection, on the other, may differ. The fact that the decision about granting the protection can be based on different grounds than the application indicates the inherent difficulties for states to correctly distinguish between seekers’ status under the Refugee Convention and based on other grounds.

With this background information, it is possible to analyse now whether asylum-seekers should be classified as some of the groups of foreigners discussed in Section II.

### B. Asylum-seekers in the Charter

How should asylum-seekers be classified in the Charter’s system? As explained above, to be regarded as group 9—migrants fully covered by the scope of the Charter—there are two general conditions: being a lawful resident or regular worker and a citizen of a state party. Seeking asylum may often signify that a person is lawfully staying within the country. However, as discussed in Section II, the European Social Charter distinguishes between lawful residence and lawful presence on the territory of a state party to the Charter. The autonomous definition of lawful residence is not provided, and it may be assumed that the Charter relies on the national understanding of what residency is.

Without a residency definition and possible reference to domestic law, the problems of interpretation of the provisions of the Charter with regard to asylum-seekers may yet occur. Many European states adopt several definitions of residency, such as for tax purposes, social security, population registration, and migration law.^43^ These definitions presuppose different lengths of lawful stay and put forward other requirements.^44^ Asylum-seekers in some countries, like Sweden, are not considered to fall within the social security and population registration notions of residency. Various views on what residency is in different states, and sometimes multiple meanings within one state party, may lead to significant confusion as to the definition that shall be taken to satisfy the requirements of the Charter. It may be obscured whether a specific group of persons, such as asylum-seekers, qualify as a resident in the ESCr meaning. It may also be unclear which of the definitions best fits the Charter. The distinction between residence and lawful stay in the Charter may point against the migration law definition, especially taking into account the history of its creation. As mentioned in Section II, the Charter was designed for state parties’ residents to ensure reciprocity between states’ social insurance systems. Similarly, the tax law and population registration definitions were not designed to answer the question of the applicability of the Charter. The social security definition might often provide lesser protection and does not always answer the question of access to healthcare services.^45^ The lesser protection may contradict the treaty’s aims, such as those stated in Part I paragraphs 11 and 13, namely, everyone’s enjoyment of the highest possible standard of health that is attainable and medical assistance for those in need.

The definition of regular work appears to be similarly obscured. National and EU law have developed autonomous interpretations of what qualifies as employment.^46^ However, the substance of the term has not yet been addressed by the Committee. The regularity of work can mean, among other things, a job obtained for a certain prolonged period, a job with fixed work time, or lawful work.^47^ Moreover, the Appendix requires regularly working within the state party’s territory.^48^ However, it is silent about where the employer shall be situated. Some asylum-seekers may retain employment in other states and work digitally from home.^49^ Whether such workers qualify for protection in the Charter is unclear so far, and the Committee has not made a stand on this issue.

As to the second condition, being a member of a state party to the Charter, not all asylum-seekers are citizens of another state party to the ESCr. The EU statistics for 2019–2022 indicate that among the thirty most common countries from which citizens seek asylum in other states, only five (Albania, Georgia, Moldova, Turkey, and Ukraine) signed and ratified the ESCr.^50^ Some asylum-seekers might not have citizenship and be regarded as stateless persons under the Convention on the Status of Stateless Persons.

Thus, it is questionable whether asylum-seekers may fall within group 9 of foreigners that receive the complete protection of the Charter for two reasons: first, their status as residents or regular workers is likely to depend on national regulation. Secondly, asylum-seekers are often not citizens of state parties to the ESCr. However, those asylum-seekers that are stateless persons (group 8) fall within the scope of the Charter and are entitled to the rights to health and medical assistance.

On several occasions, the Committee suggested considering asylum-seekers as refugees in the meaning of the Refugee Convention.^51^ The reasoning behind this is that the Refugee Convention does not connect the definition of refugees with obtaining from authorities-specific documents confirming the status of refugees or other confirmation of the status. Due to this, the Committee recognises that asylum-seekers shall receive the same right to health and medical assistance as nationals.^52^

In some cases, the Committee’s reasoning concerning recognising asylum-seekers as refugees (group 7) is in line with ordinary meaning. It thus entitles them to the complete protection of the Charter. However, as explained above in this section, a person can apply for asylum based on the Refugee Convention, national law or subsidiary grounds under the EU Qualification Directive. Those who seek asylum based on the subsidiary grounds in the Qualification Directive, such as indiscriminate violence (for example, during the war in Syria), or other reasons laid down in the national law (because of having certain diseases treatable in the host country), do not necessarily fall within the definition of refugees as provided in the Appendix to the ESCr. Therefore, the literal interpretation indicates that the Charter might not apply to this group. However, suppose such asylum-seekers are nationals of a state party and are lawfully staying on the territory of a state (group 6). In that case, they may be entitled to the right to medical assistance under Article 13(4) of the ESCr.

As mentioned above, seeking asylum often means a person is lawfully staying in a country. Can asylum-seekers be seen as irregular migrants (group 1)? If the national definition of lawful stay is to be used, national legal systems sometimes do not recognise all applications as valid. For instance, when the application was submitted after the deportation decision entered into force or after the asylum application was submitted in another EU country, some legal systems may recognise asylum-seekers as irregular within their jurisdiction.^53^

To sum up, the discussion in Section IV indicates that asylum-seekers status in the Charter is ambiguous. These groups are not homogenous. Some asylum-seekers may be regarded as, refugees and, occasionally, stateless persons. However, because the reasons for seeking asylum are no longer confined to those in the Refugee Convention, the literal interpretation of the Charter creates several sub-groups of asylum-seekers to whom the Charter applies differently, depending on the reasons for seeking asylum, citizenship or lack thereof. Here, the meeting of migration law with the social rights treaty creates a challenge for the interpretation of the latter.

## V. POSSIBILITIES FOR INCLUSION TO THE PERSONAL SCOPE

The design of the ESCr embeds certain inequalities for different groups of persons, depending on their citizenship and migration status. This section will study the existing possibilities of inclusion to the scope of the Charter to those not fully included (such as groups 1, 2, and 6).

Let’s first return to the Appendix of the ESCr. The Appendix suggests an ‘expansion clause’: the states may expand the personal scope of the treaty to the foreigners if the states so desire. The text does not specify in what form the ‘expansion clause’ can be activated; therefore, various forms can be accepted. For instance, the states may enact domestic legislation that establishes specific rights for foreigners otherwise excluded or declare their intentions internationally. The Committee considered that states might expand the personal scope, in particular, by entering other universally applicable treaties, such as the Convention for the Protection of Human Rights and Fundamental Freedoms (ECHR) or the United Nations Convention on the Rights of the Child.^54^ Interestingly, references to the United Nations Covenant on Economic, Social and Cultural Rights, which provides universal access to the right to health, are not found in the case law concerning expanding personal scope.

Another critical ‘opening’ for broadening the personal scope of the Charter is the teleological interpretation of the ESCr. The Committee regards the Convention’s purpose as ‘to give life and meaning in Europe to fundamental social rights of all human beings’.^55^ The Committee considers that the Charter should be seen as a ‘living instrument’, meaning that its substance may expand in light of present-day development.^56^

Using these two expansion possibilities, the Committee found that the normative scope of Articles 11 and 13(1) of the ESCr may apply to other groups otherwise excluded from the Charter.^57^ In the cases concerning the right to medical assistance for irregular migrants, the Committee suggests that the states have a core obligation—to deliver the necessary care to cope with immediate needs connected with human dignity, physical integrity, and the right to life—to everyone.^58^ The Committee urges the states not to interpret the immediate needs all too narrowly (as strictly urgent).^59^ This reasoning can be explained by considering that human dignity and physical integrity can go beyond the emergency care immediately necessary to save lives.

According to the Committee, Article 11 ESCr complements the right to life (Article 2 ECHR) and freedom from torture, inhumane or degrading treatment (Article 3 ECHR). The ESCr and the ECHR create a ‘normative partnership’ underlying the principle of human dignity, where healthcare is one of the prerequisites for human dignity. Furthermore, in the context of reproductive and sexual rights and environmental hazards, the Committee also refers to the right to private life (Article 8 ECHR) to establish the substance of the minimum obligations under Article 11 ESCr.^60^ However, references to Article 8 of the ECHR are rare in case law concerning migrants. There, references to Articles 2 and 3 of the ECHR are usually provided.

Since the Committee’s jurisprudence on migrants’ rights in healthcare refers to the right to life and dignified living, it is essential to understand what kind of positive substantive obligations Articles 2 and 3 of the ECHR impose. Positive substantive obligations under Article 3 of the ECHR arise when medical care is not provided at all, or the regulatory framework fails to adequately protect the patient’s life or dignity.^61^ The threshold for application of Article 2 ECHR on the right to life is relatively high: the obligations exist when a person is not necessarily in an emergency situation but can be close to it.^62^ Such situations include leaving a person without supervision close to suicide,^63^ not administering insulin to a person with diabetes,^64^ insufficient medical assistance to a person with HIV,^65^ or not providing emergency care.^66^

The threshold is also high for the qualification of Article 3 of the ECHR: it should reach the minimum level of severity. The severity level depends on the overall assessment, which includes, among other things, the degree of mental and physical suffering and other circumstances that make a victim particularly vulnerable.^67^ The European Court of Human Rights gave many examples in healthcare where a minimum threshold was reached. Such includes unsanitary conditions for a prolonged time,^68^ compulsory psychiatric care for 13 years in a language the person did not understand,^69^ denial of accessible and effective drug substitution therapy in the place of deprivation of liberty,^70^ and denying the possibility of abortion knowing that giving birth would lead to patient’s blindness.^71^ Many examples cannot be considered emergency care but rather specialised care. However, Articles 2 and 3 of the ECHR set a relatively high threshold for their application, often connected with the risk of death, mental or physical anguish, or disability.

Therefore, systematic interpretation in light of the ECHR and teleological interpretation extends states’ obligations concerning otherwise excluded groups. However, the scopes of obligations under Articles 11 and 13 are limited to saving life and dignity.

Another arguable ‘expansion’ in the Charter can be the non-discrimination framework since equality and solidarity are often seen as core values of the Charter.^72^ Freedom from discrimination is a substantive right established in article E of the ESCr.^73^ ‘National extraction’ is seen as one of the prohibited grounds for discrimination in the Charter. This non-discriminatory provision is not autonomous: the prohibition of discrimination can only be declared in conjunction with other rights laid down in the ESCr, which in our case are Articles 11 and 13 of the ESCr.^74^ As opposed to other substantive rights, such as the right to health or medical assistance, there is no possibility to opt-out from the provisions on non-discrimination, which indicates the link with the purpose of the ESCr. However, Appendix similarly applies to Article E and other substantive rights, meaning Article E is not universally applicable to all people. The Appendix draws the same distinction between different groups of foreigners as described in Section II. The teleological interpretation—that non-discrimination should apply notwithstanding migration status because equality is a core value of the ESCr—has not been extensively used by the Committee.^75^

The practice of the Committee may suggest yet another possibility for the inclusion of excluded groups to the Charter’s personal scope. This possibility can be illustrated with the help of the case of the *European Roma Rights Centre (ERRC) v Italy* (Collective Complaint No 27/2004). The case concerned Roma; some of these Roma were citizens of Italy, whereas others were irregular migrants. The Committee’s decision concerned all Roma since the distinction between those whose protection is granted and those to whom it is not was not practical to make.^76^ Similarly, it can be claimed that making the difference between different groups of asylum-seekers is impractical. There are several reasons for it. First, national healthcare legislation rarely distinguishes between different entitlements for asylum-seekers depending on the grounds for international protection. Moreover, as mentioned in Section II, the grounds for seeking protection and granting asylum do not necessarily coincide, making the distinction rather technical and impractical. The distinction between the groups of asylum-seekers that are nationals of the states to the ESCr, stateless asylum-seekers, and asylum-seekers is also rarely made in domestic legal systems, which mostly focus on access to health services for asylum-seekers as one group.^77^ Within the collective complaint procedure, the Committee will likely decide about asylum-seekers as one group.

To sum up, those foreigners classified as groups 1 (irregular migrants), 2 (non-nationals of state parties), and 6 (lawfully staying nationals of a state party) have several possibilities to receive fuller protection from the Charter. Several ‘openings’ were identified. First, the Appendix indicates that states can decide to expand the scope of the Charter by providing broader protection of the rights in national legislation or by entering an international agreement. This emphasises the subsidiary nature of the Charter with regard to domestic law and provides states leeway in deciding. The second ‘opening’ is the teleological interpretation of the Charter. The Committee considers that protection shall be afforded if the question is connected to the ‘core’ values, such as the right to life, physical integrity, and human dignity. This means that states must provide and/or compensate care when a person is likely to die or suffer (mentally or physically) significantly without it. Yet, the threshold for activating such obligations is relatively high. Finally, if making a distinction between various groups of asylum-seekers is impractical, the ECSR’s critique is likely to concern asylum-seekers as one group.

The ‘openings’ available for otherwise excluded groups have a limited scope: they are confined to serious threats to life and dignity.^78^ This limitation appears to be problematic from the public health perspective. Here, domestic laws limiting access to healthcare and the ESCr supporting this approach can be seen as a negative determinant for asylum-seekers’ health, increasing health inequalities.

## VI. CONCLUSIONS AND THE WAY FORWARD

The discussion shows that the design of the ESCr and Appendix create nine groups of foreigners, for which the applicability of the treaty differs. Such configuration of the ESCr had a purpose, in particular, not to disturb the national insurance models. Six of those groups are relevant to this article. Stateless persons (group 8), refugees as defined by the Refugee Convention (group 7) and those that are either lawful residents or regular workers and are nationals to party to the Charter (group 9) have complete access to the rights to health and medical assistance under the Charter. Those that do not fall within the definition of the Refugee Convention or are not stateless but lawfully staying and are citizens of a state party (group 6) are entitled to a limited ‘package’ of the rights: they are explicitly guaranteed the right to medical assistance. However, the enjoyment of the right to health is limited for this group to minimum obligations. These obligations usually involve saving a person’s life or human dignity. Finally, for those not citizens of a state party to the Charter (group 2) or irregular migrants (group 1), the ESCr applies only to the extent related to the minimum obligations associated with saving a person’s life or human dignity.

Yet, the domestic and EU law create new migrant statuses. The ESCr system produces certain ambiguities as to whether asylum-seekers can be seen as persons enjoying the total protection of the Charter (group 9). This ambiguity is generated due to the absence of a definition of lawful residence (compared with lawful stay) and regular work. It appears that the Committee has not attempted to provide autonomous definitions of these notions; defining them is readdressed to national legal systems. Yet, national laws may contain multiple definitions of residency for various purposes, which adds a level of unclarity to the legal status of these vulnerable groups. It also gives states leeway not to make the Charter applicable to foreigners. Similarly, the definition of regular work is uncertain. It may refer to a specific period while a person shall be employed, a work reoccurring during certain periods, any lawful occupation, etc. Therefore, whether asylum-seekers that do not fall within the definition of the Refugee Convention can receive the total protection of the ESCr (as group 9) is unclear and depend on national legislation.

Although the Committee has occasionally viewed asylum-seekers as refugees, such interpretation may depend on why persons sought international protection. Those seeking asylum based on the Refugee Convention are qualified as refugees in the ESCr meaning. However, the groups that seek asylum based on subsidiary grounds under EU legislation or other reasons established in the national law cannot be considered a refugee in the meaning of the Refugee Convention. Therefore, certain asylum-seekers cannot receive protection as refugees (group 7) in the ESCr. Depending on their citizenship, or lack thereof, they can be qualified as stateless persons (group 8), lawfully staying nationals of state parties with a right to medical assistance (group 6), as non-citizens of state parties (group 2) with only core obligations guaranteed to them. Occasionally, asylum-seekers can be also seen as irregular migrants (group 1) when the national law establishes additional thresholds for their lawful status. Therefore, the interaction of domestic law with the ESCr promotes civic stratification not only between citizens and non-citizens but also between different groups of asylum-seekers, depending on their citizenship and reasons for seeking asylum.^79^

In the article, the differences in access to rights are studied regarding healthcare services for adults. It is shown that the right to health is constructed broadly, guaranteeing the availability, accessibility, acceptability, and quality of medical services. It is emphasised that the groups included in the personal scope shall receive the same protection of the right to health as nationals. The right to medical assistance focuses on the economic accessibility of medical services. The minimum obligations signify that protection shall be given against very serious dangers to a person’s life or dignity.

Synthesizing these dichotomies mean the following in practice. Suppose the abovementioned groups of migrants would like to access vaccination against new diseases or screening for cancer. If they are classified as groups 7–9, they are supposed to be guaranteed access to this medical intervention to the same extent as nationals. However, if these foreigners are considered group 6 (lawfully staying nationals of a state party), they are not guaranteed available vaccines or cancer screenings on an equal basis with others. Similarly, states are not under obligation to raise awareness about vaccines or cancer via educating about them in languages they understand. In that case, the ESCr only guarantees the possibility of making vaccines or screenings economically accessible, for instance, when there is a confirmation that vaccination or screening is necessary due to a person’s medical conditions (such as being in a risk group). If asylum-seekers are classified as group 2 (due to the absence of nationality of a state party) or group 1 (due to their irregular status), they are not guaranteed any rights related to vaccination or screenings because this procedure is not immediately connected with protecting life or dignity. Similar reasoning can be provided concerning other non-life-saving medical services. However, if a direct threat to life or integrity exists, such as due to excruciating pain, diagnosed organ failure or life-threatening cancer, access to affordable medical services must be guaranteed to fulfil the minimum obligations. Such a design of the Charter may reinforce certain public health inequalities, civic, and social stratification.^80^

The article also discusses several possibilities for expanding the Charter’s scope to otherwise excluded groups (such as groups 1, 2 and in with regard to the right to health, group 6). One such expansion—when the protection of human rights is necessary due to considerations of human dignity—has been elucidated. As explained above, this ‘opening’ protects when life is endangered or a person endures severe suffering but does not provide protection in the situation of less immediate hazards to health. Two additional possibilities exist. First, the Committee may find distinguishing between different groups of asylum-seekers impractical and choose these groups as one. The difficulties distinguishing between different groups of asylum-seekers are obvious on the national level: those that apply based on the Refugee Convention, national or EU law usually receive the same status within the national systems. Moreover, the grounds for submitting the application and granting protection may differ, which makes the distinction between different reasons for seeking asylum impractical. Secondly, the national legislation can expand the personal scope when it desires to do so. Such expansion can be made via domestic legislation or international agreements, but it is up to the states to decide whether to grant such protection. Importantly, being a Member State of the conventions granting universal access to healthcare disregarding citizenship or reasons for stay, such as the United Nations Covenant on Economic, Social and Cultural Rights, has not yet been explicitly recognised by the Committee as expanding a personal scope of protection to all migrants.

Domestic laws often provide vague formulations regarding the care asylum-seekers are entitled to receive. Such are formulated as essential treatment, necessary care, healthcare for basic care needs, care that cannot wait, or care essential for living life in human dignity.^81^ It may be difficult to interpret formulations as going beyond minimal obligations in the Charter. These vague formulations, combined with the ambiguous status of asylum-seekers in the ESCr, do not entitle the groups as a whole to obtain greater access to rights in healthcare. On the contrary, asylum-seekers that should be seen as refugees or stateless persons (groups 7 and 8) and have full access to the rights do not receive the recognition of the right to healthcare services on an equal basis with nationals.

The analysis indicates that the application of the ESCr to asylum-seekers is nuanced, complex, and occasionally ambiguous. The ‘boxes’ of statuses created by domestic and European law for these groups of migrants do not fit in the ‘boxes’ that the ESCr creates. This imperfect fit results in situations where groups with a similar status domestically can receive different protection under the ESCr. As can be seen, citizenship, the national definition of residency and regular work, and grounds for seeking protection may significantly impact the scope of healthcare services persons are entitled to (Table 2). Much discretion regarding the substance of social rights guaranteed is provided to national jurisdictions, which can result in significant inequalities in accessing healthcare services for already vulnerable groups.

**Table 2.** Applicability of health-related provisions for different groups of asylum-seekers

Group number in this article	Groups of foreigners in the Charter	The basis for seeking asylum	Application of the ESCr’s provisions on healthcare
Group 1	Status: Unlawfully staying within a state party	National law/EU subsidiary grounds/Refugee Convention	Articles 11 and 13 apply only to provide care to protect from immediate threats to life or human dignity
	Citizenship: Any		
Group 2	Status: Lawfully staying or residing or regularly working	National law/EU subsidiary grounds	
	Citizenship: Non-citizens of a state party		
Group 6	Status: Lawfully staying foreigners	National law/EU subsidiary grounds	Article 13 applies (care shall be made affordable if a person lacks resources). Article 11 applies only to provide care to protect from immediate threats to life or human dignity
	Citizenship: Citizens of a state party		
Group 7	Status: Lawfully staying refugees as defined in the Refugee Convention	The Refugee Convention	Article 11 (care shall be accessible, available, affordable and of good quality) and Article 13 (care shall be affordable if a person lacks resources) of the ESCr apply
	Citizenship: Any		
Group 8	Status: Lawfully staying stateless persons as defined in the Convention on the Status of Stateless Persons	National law/EU subsidiary grounds	
	Citizenship: None		
Group 9	Status: Lawfully residing or regularly working foreigners	National law/EU subsidiary grounds	
	Citizenship: Citizens of a state party		

In future, the Committee’s case law can somewhat remedy inequalities in access to rights embedded in the Charter. First, the Committee may provide generous autonomous definitions of lawful residence and regular work. Suppose the Committee accepts the migration law definition of residence or prolonged stay as a lawful residence. In that case, this might be especially helpful in exercising rights under the Charter. However, such expansion is not particularly helpful for *non-citizens* of state parties that do not fall within the definition of refugees or stateless persons. The Committee can also provide an autonomous definition of refugees for the purposes of the Charter so that all persons applying for international protection under the Refugee Convention and based on subsidiary grounds or national law receive the same protection as refugees under the Refugee Convention. Secondly, the United Nations Covenant on Economic, Social and Cultural Rights contains a universal right to health, disregarding citizenship, may be seen as a declaration of the intent to expand the personal scope of the Charter. The Committee views the ECHR (especially Articles 2 and 3) and the United Nations Convention on the Rights of the Child as the instrument expanding the personal scope. A similar approach can be taken with other treaties extending the right to health to non-citizens. Thirdly, by using teleological interpretation more extensively, the Committee may provide a more generous interpretation of the non-discrimination clause in Article E of the Charter. In particular, discrimination is seen as a right relevant to everyone, disregarding citizenship in all the core United Nations conventions and the ECHR. Though the Committee often considers the references to other rights in other treaties as creating ‘normative partnership’, the room for using discrimination clauses more extensively appears to be unused. Similarly, there is a possibility of expanding the scope with the help of provisions on the right to privacy (Article 8 of the ECHR) in cases on migrants’ health, which has a lower application threshold than Articles 2 and 3 of the ECHR. However, as it stands, the national legislation providing full access to healthcare services to migrants appears to be the most effective instrument of protection.

## ETHICS

The Swedish Ethical Review Authority approved the research per the decision of 13 September 2022 dnr 2022-03738-01.

## Footnotes

1 These migrants are at a heightened risk of infectious diseases, post-traumatic stress disorder, anxiety, depression, ischaemic heart disease, heart attack, hypertension, stroke, and many others. World Health Organization, *Report on the Health of Refugees and Migrants in the WHO European Region* (WHO, 2018) 3ff.

2 Directive 2013/33/EU laying down standards for the reception of applicants for international protection (recast) [2013] OJ L 180, art 19.

3 For instance, the legislation of Belgium, Croatia, Denmark, Estonia, Finland, Germany, Hungary, Iceland, Ireland, Latvia, Lithuania, Malta, Portugal, Romania, Slovakia, Slovenia, and Sweden limits the access to healthcare for asylum-seekers. The restrictions are formulated somewhat different in these countries. Asylum-seekers are sometimes entitled to emergency care, essential treatment, necessary care, healthcare for basic care needs, care that cannot wait, and even care essential for living life in human dignity. Wet betreffende de opvang van asielzoekers en van bepaalde andere categorieën van vreemdelingen, ss 23–25, 30; Koninklijk besluit tot bepaling van de medische hulp en de medische zorgen die niet verzekerd worden aan de begunstigde van de opvang omdat zij manifest niet noodzakelijk blijken te zijn en tot bepaling van de medische hulp en de medische zorgen die tot het dagelijks leven behoren en verzekerd worden aan de begunstigde van de opvang; Pravilnik O standardima zdravstvene zaštite tražitelja međunarodne zaštite i stranca pod privremenom zaštitom, s 9; Udlændingeloven s 42 a; Tervishoiuteenuste korraldamise seadus s 6; Ravikindlustuse seadus s 5; Hälso- och sjukvårdslag (1326/2010) s 50; Lag om mottagande av personer som söker internationellt skydd och om identifiering av och hjälp till offer för människohandel (746:2011) s 26; Asylbewerberleistungsgesetz ss 4 and 6; 2007. évi LXXX. törvény a menedékjogról s 26; A Kormány 301/2007. (XI. 9.) Korm. rendelete a menedékjogról szóló 2007. évi LXXX. törvény végrehajtásáról s 26; Lög um útlendinga ss 3 and 33; European Communities (Reception Conditions) Regulations, s 18; Patvēruma likums s 11; Lege nr. 122 din 4 mai 2006 privind azilul în România s 17; Zakon o mednarodni zaščiti s 86; Lag (2008:344) om hälso- och sjukvård åt asylsökande m.fl. ss 5–6.

4 David Lockwood, ‘Civic Integration and Class Formation’ (1996) 47(3) British Journal of Sociology 531, 532 and 536ff; Lydia Morris, ‘Managing Contradiction: Civic Stratification and Migrants’ Rights’ (2003) 37(1) International Migration Review 74, 75 and 79ff.

5 The European Social Charter is not one document but a system of treaties. The European Social Charter was first adopted in 1961. Three additional protocols to the Charter and the new version of the treaty—the European Social Charter (revised) 1996—was further adopted. These five documents together form the European Social Charter’s system. Some states have signed and ratified specific protocols or the revised version ESCr, whereas others did not. These different choices of states mean that both European Social Charter and the ESCr are valid simultaneously.

6 Convention for the Protection of Human Rights and Dignity of the Human Being with Regard to the Application of Biology and Medicine: Convention on Human Rights and Biomedicine 1999 ETS 164; European Convention on Social and Medical Assistance 1953 ETS 14.

7 Because the ESCr binds the vast majority of the states in Europe, the article’s focus and references will only concern this treaty. Yet, most of the conclusions will be similar to the European Social Charter of 1961.

8 See eg Collected Edition of the *Travaux Préparatoires*, vol 1 (1953–1954) 17; Collected Edition of the *Travaux Préparatoires*, vol 5 (1958) 93–95.

9 See eg Collected Edition (1953–1954) (n 8) 20.

10 Colm O’Cinnéide, ‘Migrant Rights under the European Social Charter’ in Cathryn Costello and Mark Freedland (eds), *Migrants at Work: Immigration and Vulnerability in Labour Law* (OUP 2014) 288.

11 Convention relating to the Status of Refugees 1951 189 UNTS 137; Protocol relating to the Status of Refugees 1967 606 UNTS 267.

12 The Committee reiterates a general duty with for all rights to provide as favourable as a possible treatment to refugees and make measurable progress in achieving this goal within reasonable time. See eg ECSR Conclusions 2015—Statement of Interpretation—The Rights of Refugees under the Charter (8 October 2015) 2015_163_10/EN; *International Association Autism-Europe (IAAE) v France* Complaint no 13/2002 Decision on the Merits 7 November 2013, para 53.

13 See ECSR Conclusions XX-2—Statement of Interpretation—arts 13-1, 13-4 (2013) XX-2_035_03/Ob/EN; Collected Edition of the *Travaux Préparatoires*, vol 3 (1956) 653; Collected Edition of the *Travaux Préparatoires*, vol 4 (1957) 106; Collected Edition (1958) (n 8) 273.

14 See also Nikolaos A Papadopoulos, ‘Revisiting the Preamble of the European Social Charter: Paper Tiger or Blessing in Disguise?’ (2021) 22(1) Human Rights Law Review 6; CETS Explanatory Report to the European Social Charter (Revised) Strasbourg 1996 ETS 163, para 121.

15 See also ECSR Conclusions I—Statement of Interpretation—art 13 (31 May 1969) I_Ob_-47/Ob/EN where the Committee stated that the states ‘are no longer merely empowered to grant assistance as they think fit; they are under an obligation, which they may be called on in court to honour’, which appears to emphasise the nature of the right.

16
*International Commission of Jurists (ICJ) and European Council for Refugees and Exiles (ECRE) v Greece* Complaint no 173/2018 Decision on the Merits 26 January 2021, para 236; Statement of Interpretation XX-2 (n 13); *European Federation of National Organisations working with the Homeless (FEANTSA) v the Netherlands* Complaint no 86/2012 Decision on the Merits 2 July 2014, para 170.

17 ECSR Conclusions 2013—Bosnia and Herzegovina—art 13-1 (6 December 2013) 2013/def/BIH/13/1/EN; *Finnish Society of Social Rights v Finland* Complaint no 88/2012 Decision on the Merits 9 September 2014, para 110; Karin Lukas, *The Revised European Social Charter: An Article by Article Commentary* (EE Publishing 2021) 189.

18 See eg ECSR Conclusions 2017—Bosnia and Herzegovina—article 13-1 (8 December 2017) 2017/def/BIH/13/1/EN.

19
*Conference of European Churches (CEC) v the Netherlands* Complaint no 90/2013 Decision on the Merits 1 July 2014, para 105; *FEANTSA* (n 17), para 107; ECSR Conclusions 2017—Romania—art 13-1 (8 December 2017) 2017/def/ROU/13/1/EN; ECSR Conclusions 2015—Romania—art 13-1 (4 December 2015) 2015/def/ROU/13/1/EN; ECSR Conclusions 2009—Bulgaria—art 13-1 (2 January 2010) 2009/def/BGR/13/1/EN.

20
*European Roma Rights Centre (ERRC) v Bulgaria* Complaint no 46/2007 Decision on the Merits of 3 December 2008, para 44; ECSR Conclusions 2017—Italy—art 13-1 (8 December 2017) 2017/def/ITA/13/1/EN; Conclusions 2015—Romania (n 20).

21 ECSR Conclusions XXI-2—Greece—art 13-1 (6 February 2018) XXI-2/def/GRC/13/1/EN.

22 ECSR Conclusions XXI-2—Iceland—art 13-1 (7 February 2018), XXI-2/def/ISL/13/1/EN; Conclusions XXI-2—Luxembourg—art 13-1 (14 February 2018) XXI-2/def/LUX/13/1/EN; Conclusions XXI-2—United Kingdom—art 13-4 (8 December 2017) XXI-2/def/GBR/13/4/EN; Conclusions XXI-2—Germany—art 13-1 (8 December 2017) XXI-2/def/DEU/13/1/EN; Conclusions 2017—Armenia—art 13-1 (8 December 2017) 2017/def/ARM/13/1/EN.

23
*Médecins du Monde—International v France*, Complaint no 67/2011 Decision on the Merits 11 September 2012, paras 176–177; ECSR Conclusions 2017—Austria—art 13-1 (8 December 2017) 2017/def/AUT/13/1/EN.

24 ECSR Conclusions XX-2—Luxembourg—art 13-1 (6 December 2013) XX-2/def/LUX/13/1/EN.

25 ECSR Conclusions XXI-2—Greece (n 22); Conclusions 2017—Bosnia and Herzegovina (n 19).

26 ECSR Conclusions 2017—Austria (n 24).

27 The progressive nature of the obligations, rather than its immediate effects, was outlined from the very beginning of the Charter’s drafting history. See eg Consultative Assembly of the Committee on Social Questions, Working Party for the Preparation of a Draft European Social Charter, Preliminary Draft of Social Charter, submitted by the Secretariat of the Committee (19 April 1955) AS/Soc I (6) 1. On progressive realisation of the rights within the Charter, see IAAE (n 12), para 53; see also Gerard Quinn and Israel Doron, Against Ageism and Towards Active Social Citizenship for Older Persons. The Current Use and Future Potential of the European Social Charter (Council of Europe 2021) 119ff. The Committee has also recently acknowledged that art 11 ESCr should be interpreted as encompassing both negative obligations not to interfere, and positive obligations. The practice of the ECSR on the negative obligations was developed during the last decade. For instance, in 2018, the Committee emphasized that art 11 encompasses the negative obligations not to interfere disproportionally while imposing the obligation for transgender people to undergo sterilization to recognize their gender identity. *Transgender Europe and ILGA-Europe v the Czech Republic* Complaint no 117/2015 Decision on the Merits 15 May 2018, paras 79–89.

28 Transgender Europe and ILGA-Europe (n 28), para 71.

29 See eg *International Federation of Human Rights Leagues (FIDH) v Greece* Complaint no 72/2011 Decision on the Merits 23 January 2013, para 127; *European Committee for Home-Based Priority Action for the Child and the Family (EUROCEF) v France* Complaint no 114/2015 Decision on the Merit 24 January 2018, paras 152 and 154; *International Federation for Human Rights (FIDH) v Ireland* Complaint No 110/2014 Decision on the Merits 12 May 2017, para 139; Matti Mikkola, ‘Right to Health as a Human Right in Europe’ in Elisabeth Rynning and Mette Hartlev (eds), *Nordic Health Law in a European Context: Welfare State Perspectives on Patients’ Rights and Biomedicine* (Martinus Nijhoff Publishers 2012) 38.

30 Conclusions I (n 16).

31 The Committee explicitly states that the right to health encompasses the right to access healthcare services to everyone. *ICJ* (n 17), para 218; *Transgender Europe and ILGA-Europe* (n 28), para 71; *European Roma and Travellers Forum (ERTF) v Czech Republic* Complaint no 104/2014 Decision on the Merits 17 May 2016, para 127.

32 Conclusions I (n 16); ECSR Conclusions 2017—Russian Federation—art 11-1 (8 December 2017) 2017/def/RUS/11/1/EN.

33 ECSR Conclusions 2005—Statement of interpretation—art 11 (30 June 2005) 2005_Ob_1-1/Ob/EN; Conclusions XIX-2—Slovak Republic—art 11-1 (2 January 2010) XIX-2/def/SVK/11/1/EN.

34
*Médecins du Monde—International* (n 24), para 152; *International Centre for the Legal Protection of Human Rights (INTERIGHTS) v Croatia* Complaint no 45/2007 Decision on the Merits 30 March 2009, paras 43 and 54; ECSR Conclusions 2003—Italy—art 11-1 (30 June 2003) 2003/def/ITA/11/1/EN; Conclusions 2017—Georgia—art 11-2 (8 December 2017) 2017/def/GEO/11/2/EN.

35 ECSR Conclusions 2017—Lithuania—art 11-1 (8 December 2017) 2017/def/LTU/11/1/EN; Conclusions 2009—Ukraine—art 11-1 (2 January 2010) 2009/def/UKR/11/1/EN; Conclusions XIX-2 - Slovak Republic (n 34).

36 ECSR Conclusions 2017—Serbia—art 11-1 (8 December 2018) 2017/def/SRB/11/1/EN; Conclusions XIX-2—Latvia—art 11-1 (2 January 2010) XIX-2/def/LVA/11/1/EN; Conclusions 2009—Georgia—art 11-1 (2 January 2010) 2009/def/GEO/11/1/EN; Conclusions XXI-2—Germany—art 11-1 (8 December 2017) XXI-2/def/DEU/11/1/EN; Conclusions 2017—Turkey—art 11-1 (8 December 2017) 2017/def/TUR/11/1/EN; Conclusions I (n 16); *ICJ* (n 17), paras 225–27.

37
*Transgender Europe and ILGA-Europe* (n 28), para 79; ECSR Conclusions XXI-2—Luxembourg—art 11-1 (26 March 2018) XXI-2/def/LUX/11/1/EN; Conclusions 2017—Romania—art 11-1 (8 December 2017) 2017/def/ROU/11/1/EN.

38 This fact seems to indicate that the reasoning of the Committee on the right to health is aligned with the UN Committee on Economic, Social and Cultural Rights interpretation of the right to health. Committee on Economic, Social and Cultural Rights General Comment No 14: The Right to the Highest Attainable Standard of Health (art 12) 11 August 2000, para 12; *ERTF* (n 32), para 20; *European Roma Rights Centre (ERRC) v Bulgaria* Complaint no 151/2017 Decision on the Merits 5 December 2018, para 26; *Confederazione Generale Italiana del Lavoro (CGIL) v Italy* Complaint no 91/2013 Decision on the Merits 12 October 2015, para 69; *International Planned Parenthood Federation—European Network (IPPF EN) v Italy* Complaint no 87/2012 Decision on the Merits 10 September 2013, para 38.

39 Convention relating to the Status of Refugees 1951 189 UNTS 137; Protocol relating to the Status of Refugees 1967 606 UNTS 267, art 1A.

40 UN High Commissioner for Refugees (UNHCR), Guidelines on International Protection No 12, (2 December 2016) HCR/GIP/16/12, paras 1, 7, and 9.

41 Directive 2011/95/EU on standards for the qualification of third-country nationals or stateless persons as beneficiaries of international protection, for a uniform status for refugees or for persons eligible for subsidiary protection, and for the content of the protection granted [2011] OJ L 337/9.

42 See eg Vladislava Stoyanova and Eleni Karageorgiou, *Hur gemensamma är de gemensamma EU-reglerna? Svensk asylrätt i europeiskt perspektiv* (Delmi Policy Brief 2022:6, 2022) 41–47.

43 Jana Tepperová, ‘Personal Income Tax and Social Security Coordination in Cross-Border Employment—a Case Study of the Czech Republic and Denmark’ (2019) 21 European Journal of Social Security 23, 29–30.

44 See eg Thomas Erhag, ‘Under Pressure?–Swedish Residence-based Social Security and EU Citizenship’ (2016) 18 European Journal of Social Security 207, 214–16; Ellika Sevelin, ‘Är du bosatt lille vän? Om bosättningsbegreppet i skatte-, socialförsäkrings- och folkbokföringsrätten’ (2021) 4 Förvaltningsrättslig tidskrift 723, 726ff.

45 For example, in Sweden healthcare legislation refers to the definition provided for population registration purposes, and not social security definition. See, Hälso- och sjukvårdslag (2017:30) [Health and Medical Care Act 2017] ch 8, para 1.

46 See eg C-456/02 *Michel Trojani v Centre public d’aide sociale de Bruxelles (CPAS)* [2004] ECR I-07573.

47 ‘Regular’ (meaning 3, 1) (*Oxford Learner's Dictionaries*, 2023) <https://www.oxfordlearnersdictionaries.com/definition/english/regular_1?q=regular> accessed 4 May 2023; ‘Irregular’ (meaning 1) (*Merriam-Webster*, 2023) <https://www.merriam-webster.com/dictionary/irregular meaning 1> accessed 4 May 2023; see also, ‘Irregular and/or precarious employment’ (*European Institute for Gender Equality*, 2023) <https://eige.europa.eu/thesaurus/terms/1268> accessed 4 May 2023; Michael Jandl and others, *Migration and Irregular Work in Austria: A Case Study of the Structure and Dynamics of Irregular Foreign Employment in Europe at the Beginning of the 21st Century* (Amsterdam University Press 2009) 9–10.

48 Appendix to the European Social Charter (revised) 1996 ETS 163, para 1.

49 See eg ‘Digital refugee livelihoods and decent work—Towards inclusion in a fairer digital economy [Full report]’ (*ILO*, 29 April 2021) <https://www.ilo.org/global/topics/labour-migration/publications/WCMS_785236/lang–en/index.htm> accessed 4 May 2023.

50 Eurostat, Annual asylum statistics (2023) <https://ec.europa.eu/eurostat/statistics-explained/index.php?title=Annual_asylum_statistics > accessed 17 April 2023.

51 ‘Annual asylum statistics’ (*Eurostat*, 28 April 2023) <https://ec.europa.eu/eurostat/statistics-explained/index.php?title=Annual_asylum_statistics> accessed 4 May 2023.

52
*ICJ* (n 17), para 80; ECSR Conclusions 2015 (n 12).

53 See e.g. Utlänningslagen (2005:716) Kap 5 s 1 b.

54 ECSR Conclusions 2004 - Statement of Interpretation—Personal Scope of the Charter (2004) 2004_163_01/Ob/EN; *Defence for Children International (DCI) v Belgium* Complaint no 69/2011 Decision on the Merits 23 October 2012, paras 29, 31; *CEC* (n 20), paras 68–69; see also Matti Mikkola, ‘Social Human Rights of Migrants under the European Social Charter’ (2008) 10 European Journal of Social Security 25, 37.

55
*EUROCEF* (n 30), para 53; CEC (n 20), para 67.

56
*EUROCEF* (n 30), para 53; *DCI* (n 55), para 30; *Federation of Human Rights Leagues (FIDH) v France* Complaint no 14/2003 Decision on the Merits 8 September 2004, paras 27 and 29.

57 Conclusions 2005 (n 34); *ICJ* (n 17), para 220; *Transgender Europe and ILGA-Europe* (n 28), para 73; *IPPF EN* (n 39), para 66.

58
*ICJ* (n 17), para 236. Conclusions XXI-2—Greece (n 22), *CEC* (n 20), para 73; *FEANTSA* (n 17), para 173.

59
*CEC* (n 20), para 105; Conclusions XXI-2—Greece (n 22); Conclusions XX-2 (n 13).

60
*Transgender Europe and ILGA-Europe* (n 28), para 83; Conclusions 2021—Romania—art 11-1 (31 December 2021) 2021/def/ROU/11/1/EN.

61
*Lopes de Sousa Fernandes v Portugal* [GC] App no 56080/13 (ECtHR, 19 December 2017, paras 173–74, 187–88.

62
*Osman v the United Kingdom*, [GC] ECHR 1998-VIII, para 116; see also Alastair Mowbray, The Development of Positive Obligations under the European Convention on Human Rights by the European Court of Human Rights (Hart Publishing 2004) 15–17; David Harris, Michael O’Boyle, Ed Bates, and Carla Buckley (eds), Harris, O’Boyle & Warbrick: Law of the European Convention on Human Rights (OUP 2018) 209.

63
*Fernandes de Oliveira v Portugal* [GC] App no 78103/14 (ECtHR, 31 January 2019), para 115.

64
*Aftanache v Romania* App no 999/19 (ECtHR, 26 May 2020), para 53.

65
*Kats and Others v Ukraine* App no 29971/04 (ECtHR, 18 December 2008), paras 107–112.

66 See eg *Lapshin v Azerbaijan* App no 13527/18 ECtHR, 20 May 2021), para 72, *Plokhovy v Russia* App no 45024/07 (ECtHR, 22 December 2020), paras 81–86; *Karakhanyan v Russia* App no 24421/11 (ECtHR, 14 February 2017), paras 47–50.

67
*Rooman v Belgium*, [GC] App no 18052/11 (ECtHR, 31 January 2019), para 141; Natasa Mavronicola, Torture, Inhumanity, and Degradation under Article 3 of the ECHR: Absolute Rights and Absolute Wrongs (Hart Publishing 2021) 90.

68
*Stanev v Bulgaria*, [GC] App no 36760/06 (ECtHR, 17 January 2012), paras 210–11.

69
*Rooman* (n 68) para 141.

70
*Wenner v Germany*, App no 62303/13 (ECtHR, 1 September 2016), para 55.

71
*Tysiąc v Poland*, ECHR 2007-I, para 67.

72 ECSR Conclusions XX-4 - Statement of Interpretation—The Rights of Refugees under the Charter (8 October 2015) XX-4_035_08/EN.

73 CETS, Explanatory Report to the European Social Charter (Revised) Strasbourg 1996 ETS 163, para 136; *FIDH v Ireland* (n 30), para 122; Lukas (n 18) 341.

74
*IAAE* (n 12), para 51; *International Federation of Associations of the Elderly (FIAPA) v France* Complaint no 145/2017 Decision on the Merits 11 May 2019, para 43; *Confédération française de l'encadrement CFE-CGC v France* Complaint no 56/2009 Decision on the Merits 23 June 2010, para 81.

75
*DCI v Belgium* (n 56), para 150; *Defence for Children International (DCI) v The Netherlands* Complaint no 47/2008 Decision on the Merits 20 October 2009, paras 73–75. See the exception in *European Roma and Travellers Forum (ERTF) v France* Complaint no 64/2011 Decision on the Merits 24 January 2012, paras 127–129.

76
*European Roma Rights Centre (ERRC) v Italy* Complaint no 27/2004 Decision on the Merits 7 December 2005, para 18.

77 See (n 3).

78
*ICJ* (n 17), para 82; *EUROCEF* (n 30), para 53; *DCI* (n 55), para 35; *FIDH v France* (n 57), para 30; *FEANTSA* (n 17), para 59.

79 Lydia Morris, *Asylum, Welfare and the Cosmopolitan Ideal: A Sociology of Rights* (Routledge 2010) 112–13.

80 On the need to consider political (legal) determinants of public health see eg Jacqueline M Torres and Roger Waldinger, ‘Civic Stratification and the Exclusion of Undocumented Immigrants from Cross-border Health Care’ (2015) 56(4) Journal of Health and Social Behavior 438, 451.

81 See (n 3) above.

